# Timing and quality of sleep in a rural Brazilian family-based cohort, the Baependi Heart Study

**DOI:** 10.1038/srep39283

**Published:** 2016-12-23

**Authors:** F. Beijamini, K. L. Knutson, G. Lorenzi-Filho, K. J. Egan, T. P. Taporoski, L. K. G. De Paula, A. B. Negrão, A. R. V. R. Horimoto, N. E. Duarte, H. Vallada, J. E. Krieger, M. Pedrazzoli, A. C. Pereira, M. von Schantz

**Affiliations:** 1 Institute of Psychiatry, University of São Paulo Medical School, São Paulo, SP, Brazil; 2 Centre for Health Sciences, State University of Western Paraná, UNIOESTE, Francisco Beltrão, PR, Brazil; 3 Department of Medicine, University of Chicago, Chicago IL, USA; 4 Sleep Laboratory, Pulmonary Division, Heart Institute (InCor) do Hospital das Clínicas da Faculdade de Medicina da Universidade de São Paulo, SP, Brazil; 5 Faculty of Health and Medical Sciences, University of Surrey, Guildford, Surrey, UK; 6 Laboratory of Genetics and Molecular Cardiology, Heart Institute (InCor), University of São Paulo Medical School, São Paulo, SP, Brazil; 7 School of Arts, Sciences and Humanities, University of São Paulo, SP, Brazil

## Abstract

Sleep is modulated by several factors, including sex, age, and chronotype. It has been hypothesised that contemporary urban populations are under pressure towards shorter sleep duration and poorer sleep quality. Baependi is a small town in Brazil that provides a window of opportunity to study the influence of sleep patterns in a highly admixed rural population with a conservative lifestyle. We evaluated sleep characteristics, excessive daytime sleepiness, and chronotype using the Pittsburgh Sleep Quality Index, Epworth Sleepiness Scale and Morningness-Eveningness Questionnaire questionnaires, respectively. The sample consisted of 1,334 subjects from the Baependi Heart study (41.5% male; age: 46.5 ± 16.2 y, range: 18–89 years). Average self-reported sleep duration was 07:07 ± 01:31 (bedtime 22:32 ± 01:27, wake up time: 06:17 ± 01:25 hh:min), sleep quality score was 4.9 + 3.2, chronotype was 63.6 ± 10.8 and daytime sleepiness was 7.4 ± 4.8. Despite a shift towards morningness in the population, chronotype remained associated with reported actual sleep timing. Age and sex modulated the ontogeny of sleep and chronotype, increasing age was associated with earlier sleep time and shorter sleep duration. Women slept longer and later, and reported poorer sleep quality than men (p < 0.0001). This study provides indirect evidence in support of the hypothesis that sleep timing was earlier prior to full urbanisation.

Sleep has several physiological functions relevant to health. Several reports have suggested that poor sleep quality, short sleep duration, or inappropriate sleep timing are related to cardiovascular diseases (CVD)^1^, metabolic dysfunction^2^, immune dysregulation^3^ and cognitive impairment^4^. Sleep is known to be intimately regulated by circadian processes^5^. Consequently, the exposure to natural light-dark cycle is fundamental to the entrainment of circadian system and sleep-wake cycle^6^. However, sleep patterns and habits are also intimately associated with cultural and socio-economic factors, necessitating field studies evaluating a variety of populations^7^.

It has been proposed that electricity and urbanisation have changed human sleep behaviour and sleep patterns, such as timing and duration^8^. In modern metropoles, sleep complaints have increased progressively in the past decade, with a considerable delay in bedtime and wake up times^9^. For instance, two recent studies from representative samples of adults from urban populations of Chicago and São Paulo described average sleep duration shorter than 7 h^10^. A nationwide report from the National Sleep Awareness Roundtable in the United States also indicate that 35.3% of respondents were sleeping less than 7 h^11^. Evaluating sleep in populations from regions still undergoing urbanisation is crucial for developing a better understanding and knowledge of human sleep and its natural history. Studies have been performed with religiously conservative populations^12^, and also populations with limited access to electricity. Rubber tappers in the Amazon region of Brazil, for example, studied in two different conditions with and without access to electricity, presented sleep onset and wake up times very tightly associated with environmental light, furthermore, they presented advanced sleep timing when they had no access to electricity^13^. In another study, access to electric light was associated with a reduction of sleep duration in a hunter-gatherer community in Argentina^14^. In short, several studies suggested that access to electricity has delayed sleep timing^14^,^15^,^16^, and also shortened sleep duration^14^. Recently, however, an objective evaluation of sleep duration in three hunter-gatherer societies found average sleep durations between 5.1 and 7.1 hours^17^. Furthermore, these communities also showed significant seasonal patterns, and sleep timing was more likely to be associated with environmental temperature rather than with light (sunset or sunlight). Thus, there is no current strong consensus on the outcomes of urbanization on sleep patterns, and consequentially, more data from a greater diversity of communities are needed.

The Baependi heart study is a family-based cohort study designed to investigate cardiovascular disease and associated factors, specifically factors typical of Brazil^18^. This town is located at 21.5°S 44.5°W, with a photoperiod range between 10 h 40 min and 13 h 20 min, and an average insolation ranging between 4.09 and 5.59 kWh/m^2^/day (http://www.esrl.noaa.gov/gmd/grad/solcalc/). The traditional lifestyle, moderate and ongoing urbanisation, and very limited inward migration of this rural town make it a location of interest for the study of sleep quality and sleep characteristics. Since the beginning of this study in December 2005, a significant number of discoveries have been reported, such as the heritability of cardiovascular risk factors^19^, including sedentary behaviour^20^, smoking^21^, glycaemic control and arterial stiffness^22^, and obstructive sleep apnoea^23^. Moreover, we recently reported the heritability of chronotype in this population and found that genetic factors account for 48% of chronotype when adjusted for sex and age, or 38% when adjusted for sex, age and residential zone (rural *versus* municipal)^24^. Furthermore, we demonstrated a significant shift towards morningness in the frequency distribution of chronotypes in the Baependi Heart Study population. This raised the question of whether this diurnal preference was actually associated with significantly earlier self-reported bedtimes and wake up times (as opposed to self-reported preferred ones). Here, we have combined the primary datasets underlying these reports, the Morningness-Eveningness questionnaire (MEQ)^25^, the Pittsburgh Sleep Quality Index (PSQI)^26^, and sleepiness as assessed with the Epworth Sleepiness Scale (ESS)^27^. The quantitative answers to PSQI allowed us to consider self-reported rise and bed times.

## Results

### Population, lifestyle, demographic data, and sex comparisons

Table 1 describes the demographic characteristics of the sample, as well as the sleep measures collected. The entire study sample was 1,847 subjects, out of which 1,334 individuals were considered eligible for statistical comparisons. Subjects missing data-points in one or more questionnaires were excluded from this analysis. The age range of the sample was 18 to 89 years. 31.7% of subjects presented excessive daytime sleepiness (scores equal or above 10 on the ESS), and 34.9% presented scores equal or above 5 on the Pittsburgh Sleep Quality Index (PSQI), meaning that their sleep quality was classified as poor. Women had poorer self-reported sleep quality than men based on PSQI score (means ± standard deviation) (5.4 ± 3.3 *vs* 4.5 ± 2.9; p < 0.001, Cohen’s d value = 0.18), accounting for a small difference between the sexes. Consistent with this, more women had a PSQI score above 5 than men (39.9% *vs* 27.8%, **Χ**^2^ = 20.79; p < 001). Women also presented later wake up times (means ± standard deviation (06:26 ± 01:17) *vs* (06:04 ± 01:34); p < 0.01, Cohen’s d value = 0.17), meaning that this difference between sexes was small. Women spent more time in bed than men (means ± standard deviation (07:52 ± 1:26) *vs* (07:32±01:31) hour:min ± hour:min; p < 0.005, Cohen’s d value = 0.15). Self-reported sleep duration was longer in women than men (means ± standard deviation (07:13 ± 1:33) *vs* (06:57 ± 1:27) hour:min ± hour:min; p < 0.01); however, the difference was small according to the Cohen’s d value of 0.10.

### Description of sleep quality – The PSQI and its components

**Figure 1:**
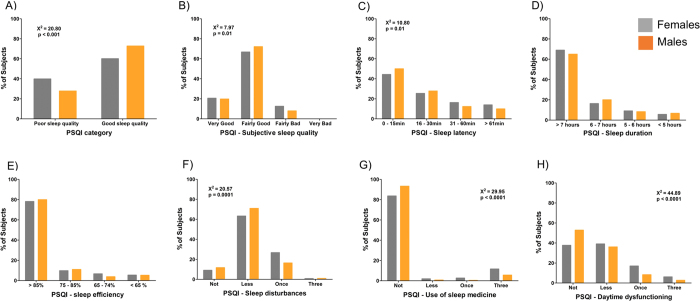
Frequency distribution for PSQI category and components by sex. (**A**) PSQI category. Subjects with scores ≥5 were classified as having poor sleep quality. A higher frequency of women presented poor sleep quality **Χ**^2^ = 20.80; p < 0.001. (**B**–**H**) show PSQI components. (**B**) Subjective sleep quality. There was a sex effect on how subjects rated their sleep, with women tending to declare poorer sleep quality **X**^2^ = 7.97; p < 0.05. (**C**) A higher proportion of women reported longer sleep latency **X**^2^ = 10.81; p < 0.05. (**D**) Sleep duration and (**E**) sleep efficiency, no statistical difference between the sexes. (**F**) Sleep disturbances, women were more prone to declare having sleep disturbances during the last month **X**^2^ = 20.37; p < 0.001; (**G**) Use of sleep medication: Men declared less use of sleep medication than women during the last month **X**^2^ = 29.95; p < 0.00. (**H**) Daytime dysfunction: A higher proportion of women declared having daytime dysfunction during the last month **X**^2^ = 44.89; p < 0.0001.

To further explore the sleep quality in this population, we first evaluated the frequency distribution of PSQI scores by sex (Fig. 1 in Supplementary files). Compared to men, a significant (**Χ**^2^ = 20.80; p < 0.001) number of women presented scores above 5 in PSQI, meaning poor sleep quality. In order to investigate the relationship between sex and sleep quality, we analysed the score of the seven different components of the PSQI: Subjective sleep quality, sleep latency, self-reported sleep duration, sleep efficiency, sleep disturbances, use of sleep medication, and daytime dysfunction. Each component is derived from one or more questions and receives a score from 0 to 3, a lower the score indicates better sleep quality. Figure 1 presents the frequency distribution for PSQI classification and PSQI components according to sex. In general, women presented higher scores (worse sleep) for most of the components with the exception of sleep duration and sleep efficiency. Importantly, out of 1,334 questionnaires received, there was not a single score of 3 for subjective sleep quality, meaning that no one evaluated their sleep as “very bad” (Fig. 1B). However, over 60% of subjects from both sexes classified their sleep as “fairly good”.

The frequency distribution analysis of PSQI categories also permit identification of the components sleep latency, sleep duration and sleep disturbances as the main contributors to high PSQI total scores. However, not a single subject described their sleep as “very bad”. Conversely, 23.3% of subjects presented sleep disturbances at least once in the past month, and 10.2% of the sample reported not having a single sleep disturbance episode. In order to identify the most prevalent sleep disturbance in this population, we evaluated the specific answers to the question regarding sleep disturbances (“*During the past month, how often you had trouble sleep because you…*”) Fig. 2 in Supplementary file presents a detailed description of the frequency of the different sources of sleep disturbance. Interestingly, the most commonly selected answers were waking up in the middle of the night, long sleep latency (>30 minutes), and having to get up to use the bathroom.

### Description of self-reported sleep patterns

**Figure 2:**
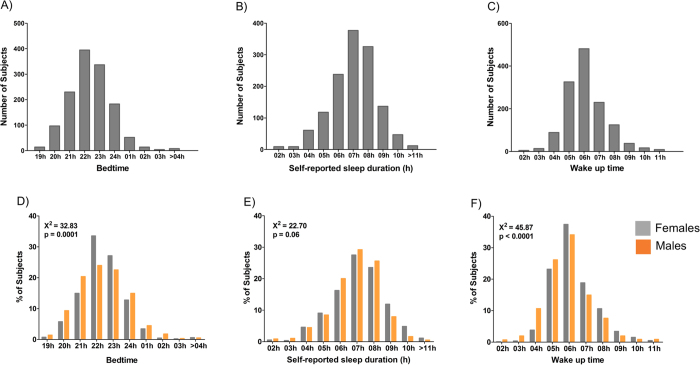
Frequency distribution histograms for self-reported sleep patterns derived from the PSQI (1,334 subjects were included). The upper panel shows the entire population, and the lower panel the same dataset divided by sex. (**A** and **D**) show bedtime frequency distribution binned by 1-hour intervals from 19 h to after 04 h. (**B** and **E**) show sleep duration frequency distribution binned by 1-hour intervals from 02 h to >11 h of sleep duration. (**C** and **E**) show wake-up time frequency distribution binned by 1-hour intervals from 01 h to 11 h. There was higher proportion of male subjects going to bed before 23 h **χ**^2^ = 32.83; p < 0.0001 (**D**), and a higher proportion of male subjects presenting earlier wake up times **Χ**^2^ = 45.87; p < 0.001 (**F**).

Next, to evaluate the sleep patterns of this population, we extracted the information from the PSQI related to the timing and duration of sleep: Bedtime, sleep duration, and wake up time. Self-reported bedtime, wake up time, and sleep duration were plotted in frequency distribution histograms, (Fig. 2). The sleep timings confirmed the “early bird” pattern of the majority of the sample with a large proportion (48%) of subjects presenting bedtimes between 22 and 23 h and wake-up times between 05 and 06 h. A large proportion (67%) of subjects also reported sleeping 7 or more hours.

The lower panel of Fig. 2 shows the frequency distributions of sleep timings according to sex. In general, a higher proportion of male subjects tended to present earlier bedtimes and wake up times (p < 0.01). Furthermore, a higher proportion of male subjects reported sleeping six to eight hours, while a higher proportion of women reporting sleep duration longer than nine hours (p = 0.06). This difference in distribution between short- and long-sleepers is obscured in the average sleep timing values in Table 1, which do not differ significantly between the sexes.

### Chronotype associations with sleep patterns, sleep quality, and sleepiness

**Figure 3:**
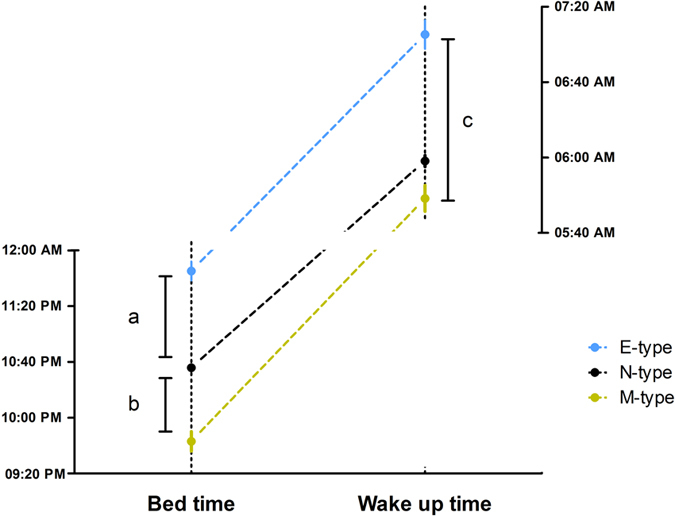
Sleep patterns according to chronotype. Means and standard error of the mean for Bed time and Wake up time extracted from the PSQI. E-types (N = 125) presented later bedtime in comparison to M-types (N = 97) and N-types (N = 830) subjects. M-types subjects also went to bed earlier than N-types. Bonferroni *post-hoc* comparisons for (**a** and **b)** produced p < 0.0001. E-types also presented later wake up time in comparison to M-types and N-types, Bonferroni *post-hoc* comparison for (**c**) yielded p < 0.0001.

We classified chronotype using a variation of the method of Robilliard *et al*.^28^. In summary, we plotted MEQ scores as a function of age. Because of the previously reported kurtosis of MEQ at higher ages, caused by the imperfect compatibility between the high general morningness of this population and the design of the scale^24^, only subjects aged less than 60 years old were included in this analysis. A linear regression analysis of age and MEQ score was performed, and subjects presenting MEQ scores 20% above the regression line were categorized as Morning type (M-type) and those 20% below the regression line were Evening type (E-type). Subjects within 20% of the regression line were categorized as Neither type (N-type). See Fig. 3 in Supplementary file for a detailed description of chronotype categorization. One-way ANOVA confirmed that subjects categorized as M-types presented earlier bedtimes and wake up times in comparison to E-types (p < 0.0001 and p < 0.0001). M-types spent on average more time in bed (08:14 ± 1:10 h) than N-types (07:40 ± 1:20 h) and E-types (07:39 ± 1:33 h) (p < 0.05; Bonferroni *post-hoc* p < 0.01). Consistent with this, M-types also reported longer sleep duration (07:46 ± 1:24) than N-types (07:07 ± 1:28) and E-types (06:59 ± 1:42 h) (p < 0.001; Bonferroni *post-hoc* p < 0.001). Figure 3 summarizes the sleep patterns for the three categories of chronotype. Regarding sleep quality measured by PSQI, E-type (5.73 ± 3.64 h) subjects presented poorer sleep quality than M-types (4.01 ± 2.62 h) and N-types (4.7 ± 3.1 h) (p < 0.001). No effect of chronotype on sleepiness was found (p = 0.076).

### Age and sex influence on sleep patterns and chronotype

To further evaluate the relationship between age and sex on sleep patterns and chronotype ontogeny, the sample was binned in 5 age groups (18–31y; 31–40y; 41–50y; 51–60y and 61 and up). Detailed information about the age groups is presented in Table S1.

**Figure 4:**
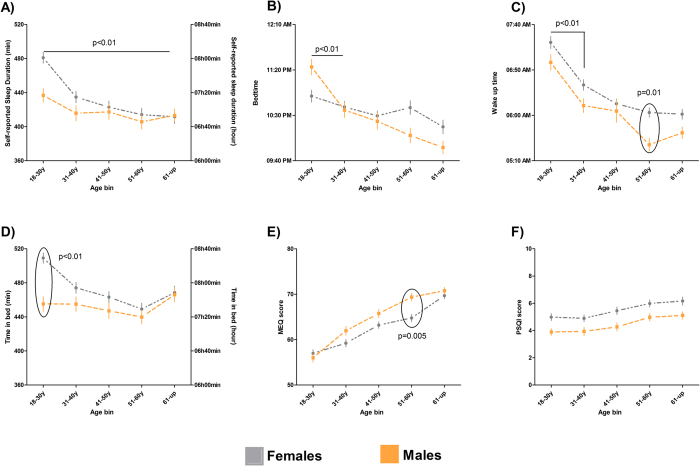
Age and sex influence sleep characteristics and chronotype. Values are presented as means ± standard error (**A**) Self-reported sleep duration. Bonferroni *post hoc* test indicated a difference between means of sleep duration at the youngest (18–30y) and the oldest age bins (61-up) for both sexes. (**B**) Self-reported bedtime. Bonferroni *post hoc* test suggested a significant advance in bedtime of men from the age bin 18–30y to the 31–40y when women presented later bedtimes. (**C**) Self-reported wake-up time, Bonferroni *post hoc* test found significant differences between the youngest (18–30 years) to the second youngest (31–40 years) bins in both sexes, with significant sex differences emerging for the age bin 51–60 years. (**D**) Time in bed. Bonferroni *post hoc* test indicated a significant difference between sexes for the youngest age bin (18–30 years). (**E**) MEQ score, Bonferroni *post-hoc* testing yielded a statistically significant difference between sexes in the age bin 51–60y, when men reported more morningness. (**F**) PSQI score, no interaction was found, women presented increasingly poor sleep quality after 41–50 years in comparison to youngest age bin (18–30 years).

In this way, we evaluated the ontogeny of chronotype and sleep patterns according to sex. Figure 4 summarises the results for comparison of self-reported sleep duration, bedtime, wake up time, time in bed, chronotype, and sleep quality (PSQI score) across age and between sexes. Sex modulated self-reported sleep duration (p = 0.002). Sleep duration also varied according to age in the population (p < 0.001). The interaction between sex and age (p = 0.02) indicated an age-related sex difference in sleep duration (Fig. 4A). Self-reported bedtime was associated with age (p < 0.0001); however, there was no effect of sex (p = 0.29). An interaction between factors was found (p = 0.003), meaning that the age effect on bedtime is modulated by sex (Fig. 4B). Young male subjects presented later bedtimes than women of the same age group, whilst older men presented earlier bedtimes than older women. Sex also modulated wake up time (p < 0.0001); women woke up later than men. Wake up time was modulated by age as well, with older subjects waking up earlier (p < 0.0001); however, no interaction between sex and age was found (p = 0.43) (Fig. 4C). Age (p < 0.0001) and sex (p < 0.0001) also modulated time in bed in an interacting fashion (p < 0.01) (Fig. 4D). Sex was found to influence chronotype (p = 0.0006), and male subjects presented on average higher MEQ scores (greater morningness) than women. Chronotype also changed as a function of age (p < 0.0001) with scores for MEQ increasing with age. There was an interaction between age and sex (p = 0.01). Women aged 51–60 years old were significantly less prone to morningness than men (Bonferroni *post hoc* test, p = 0.01) (Fig. 4E). Sex modulated sleep quality, with female subjects presenting poorer sleep quality (p < 0.0001). Older subjects also presented poorer sleep quality (p < 0.0001). No interaction was found (Fig. 4E).

Age and sex comparisons also were performed for sleepiness. Sleepiness scores did not change according to age (p = 0.076) or differ between sexes (p = 0.91); consequently, no interaction between factors was found (p = 0.09) (for means and standard error please see Table S1).

### Characterizing poor sleepers

Our initial hypothesis was that subjects from Baependi would present high sleep quality, based on consideration the mean of PSQI score for the entire sample (4.9 ± 3.2) one could accept our hypothesis. However, 34.9% of subjects were poor sleepers (scores >5 on PSQI). According to Fig. 4F, PSQI scores are subject to sex and age influences. In order to understand who the poor sleepers are in this population, we evaluated and compared sleep characteristics and chronotype of subjects classified as poor and good sleepers. Table 2 summarises our findings. E-types were significantly overrepresented amongst poor sleepers. Furthermore, poor sleepers also presented later bedtimes (p < 0.001), shorter sleep duration (p < 0.001), spent less time in bed (p = 0.01) and had longer sleep latency (p < 0.001) than subjects classified with good sleep quality.

## Discussion

We set out to provide a quantitative and qualitative description of sleep patterns, and describe in detail the relationships between age, sex, and chronotype with sleep quality and timing in this rural population. We found that the self-reported mean bedtime and wake up times were 22:32 ± 01:27 (SD) and 06:17 ± 01:25 respectively, which was consistent with the previously reported morning preference of this population. Sleep duration was 07:07 ± 01:31(hh:min). Additionally, 31.7**%** of subjects presented with excessive daytime sleepiness and the mean Epworth Sleepiness Score (ESS) was 7.8 ± 4.8, below the threshold for excessive sleepiness. However the mean of PSQI score for the entire sample was 4.9 ± 3.2 and 34.9% were categorized with poor sleep quality. Age played a role in sleep quality — the older the subjects, the poorer sleep quality. Women presented poorer sleep quality than men, but no subject ranked their sleep quality as “very bad” on the Pittsburgh Sleep Quality Index. One of the main factors influencing the total PSQI scores may be sleep latency, which also increased with age. As presented in Fig. 1C there was a high proportion of subjects reporting latencies longer than 30 minutes. Chronotype was another important factor modulating sleep quality. E-types presented poor sleep quality than M-types and N-types.

The Baependi Heart Study population presented a high prevalence of self-reported poor sleep quality. The mean PSQI score for the full sample was 4.9 (3.2), which is higher than studies elsewhere including the control group in the validation of the Brazilian version for the PSQI questionnaire^29^ (which was 2.5). In other studies, the prevalence of poor sleep was lower in reports from Japan (9.5% of total men and 14.3% of total women having PSQI ≥ 6)^30^ and China (26.36% having PSQI ≥ 6)^31^. However, the prevalence of 34.9% of poor sleepers described here is similar to what has been reported in United States^32^,^33^ and Korea^34^. Notwithstanding this point, none of 1,334 subjects included in this report classified their own sleep quality as “very bad”. The mean of subjective sleepiness reported here is comparable to what was found in other studies in less urbanized populations (9.1 ± 3.4 for men and 7.6 ± 3.2 for women)^12^, but below what has been reported in highly urbanized centres (13.1 ± 2.3 for the entire population)^34^. Thus, our findings show that poor sleep quality is not necessarily less common in a small quiet rural town with a high prevalence of physical labour (it is worth noting that access to electricity is essentially universal in Baependi). Our results also indicate a strong ontogenetic influence on poor sleep quality. The age range in the study population was 18 to 89 years of age. Men presented mean PSQI score above the threshold for poor sleep quality at the 51–60 years age bin (see Table S1), while females crossed the threshold at the age bin of 41–50 years. These age differences in sleep quality are previously known^9^,^35^,^36^ and have been described worldwide^36^.

Sex also modulated sleep quality in our study sample. Women presented poorer sleep quality than men. Post-menopausal women presented higher prevalence of sleep disturbance (higher than 40% after 50 years of age^37^) in comparison to pre-menopausal women. Insomnia is a frequent sleep problem in midlife women, with prevalence around 20%^38^,^39^. We did not evaluate specific sleep disturbances or insomnia, although we found a higher prevalence of women reporting sleep latencies longer than 30 minutes more frequently than men (see Fig. 3 in Supplementary file). Also, PSQI scores higher than 5 have been reported as good predictor for primary insomnia^40^. In this way, further evaluation of the association between menopause and sleep quality should be performed in this population. Furthermore, there can be sex differences related to the way people respond to the questionnaire. In this sense, subjective sleep quality differs between men and women in this community, but it can not be inferred from the observations made here whether the subjective sleep quality differences entirely reflect differences in objective sleep quality.

Another finding of this study was the influence of sex on ontogeny of sleep patterns (bedtime, wake up time and sleep duration). Men and women did not differ significantly in diurnal preference until age of 50 years old when men presented higher morningness. A similar pattern of chronotype ontogeny was described in a very large Brazilian study showing that, although morningness increases with age in both men and women, it does so with different slopes, so that men (who start life with higher average eveningness) have a higher average morningness tendency from age 50 onwards^24^. However, as previously described^24^, the Baependi study population presents a general shift towards morningness. This trend can be seen even for the young age bin. The mean score for the youngest age bin in Baependi study was similar to the mean score of age bin >55 years in the previous report from Brazil on the ontogeny of chronotype, which was assembled from a much larger and more metropolitan, population sample, whereas subjects aged 55 and over in Baependi had a much higher morningness^24^.

Sleep timing and sleep duration were modulated by age and sex as mentioned before. Furthermore, these variables were also strongly associated with chronotype categories. Subjects from Baependi presented considerably earlier wake up and bed times similar to what have been reported in rural populations^13^,^14^,^41^,^42^, in contrast with late sleep timing reported in metropolitan locations^9^,^43^. It has been demonstrated that urbanisation may shift chronotype frequency distribution towards greater eveningness, and a previous study from our group reported the distribution of diurnal preference in Baependi to be shifted towards morningness^24^. This raised the question whether the actual sleep patterns of the Baependi population would also be shifted to morningness. The results presented here support this hypothesis. Being exposed to a natural high-amplitude light:dark cycle has a strong influence on circadian rhythms, including entrainment of sleep timing^6^. One can hypothesize that subjects living in areas such as the Baependi region, where they are highly exposed to the natural light:dark cycle, would cause sleep schedules to be significantly more synchronized by this zeitgeber. This is supported by our finding that not only are the preferred bed time and wake times advanced, but also the reported actual ones in comparison to urban areas^9^,^35^,^43^.

This study presents strengths and limitations worth mentioning. We report subjective data based on questionnaires. Although these instruments have been used for sleep research and in sleep clinics worldwide, they contain measures that are subjected to considerable variation in the subject’s personal interpretation. The life experience of Baependi residents may be uniquely reflected in some of their answers. Once supplemented with objective measures, such as actimetry and circadian phase markers, these datasets can be combined with the multiple layers of phenotype data from the Baependi study, and be related to health outcomes and major risk factors. The findings from this interesting cohort show that the presence of electrification does not obligately (or at least not immediately) lead to shifted chronotype distributions towards eveningness and chronotype and delayed sleep and wake timings. They also indicate that an earlier sleep phase does not equate to a perception of better sleep. Taken together, they justify the further investigation of the environmental and lifestyle factors associated with these observations. The longitudinal nature of the Baependi Heart Study will make it possible to investigate whether and how these parameters will change over future years.

## Methods

The methodology for recruitment has been described in detail previously^18^. All participants were aged 18 or older and gave written informed consent before participation. This study protocol conformed to international ethics standards based on the Declaration of Helsinki and was approved by the local ethics committee (Hospital das Clínicas – Universidade de São Paulo, Brazil). This report is based on the evaluation of a sample from 95 genealogical families (as opposed to households), encompassing a total of 1,847 subjects from the Baependi Heart Study. It includes data from the second data collection wave of the Baependi study, when the sleep measures were included. The following instruments were applied as part of two booklets of questionnaires applied in two different sessions containing the following instruments: (i) the Portuguese version of Pittsburgh Sleep Quality Index (PSQI)^44^, (ii) the Epworth Sleepiness Scale (ESS) in a validated Brazilian Portuguese translation^29^ and (iii) the Morningness-Eveningness Questionnaire (MEQ)^45^ as previously described^24^.

The PSQI was developed to assess the quality and disturbances related to sleep over a period of 1 month. It is composed of seven different components: subjective sleep quality (C1), sleep latency (C2), sleep duration (C3), habitual sleep efficiency (C4), sleep disturbances (C5), use of sleeping medication (C6) and daytime dysfunction (C7). All the seven components are graded on scores from 0 to 3, and the sum of these components scores yields a global score from 0 to 21. The higher the global score, the worse the sleep quality; a score higher than 5 is associated with poor sleep quality^26^. Furthermore, the PSQI also asks subjects about their habitual sleep patterns, such as bedtime, wake up time, sleep duration, and sleep latency. The Morningness-Eveningness questionnaire aims to identify the chronotype (diurnal preference) of a person. However, to categorize subjects we used a variation of the method of Robilliard *et al*.^28^. In summary, we plotted MEQ score against age for all individuals in the study population aged between 18 and 60 years. Further, a linear regression analysis was performed and subjects away from the regression line were selected to be categorized as Morning type (20% above the regression line) or Evening type (20% bellow the regression line). Subjects in between lines were categorized as Neither type. The Fig. 3 in Supplementary file shows a detailed description for the categorization applied here. The Epworth sleepiness scale is compound by 8 situations in which subjects rate the likely to doze in ref. 27. The ESS produces a score from 0 to 24 and results equal or above 10 are considered excessive daytime sleepiness.

From the entire sample, data from 1,334 individuals were considered complete and eligible for statistical comparisons, the results of which are presented here. Data from subjects with missing one or more answers for more than one questionnaire were excluded. Data were analysed by comparison of means using Student’s t tests, one-way analysis of variance, and multiple factors analysis of variance. To evaluate distributions, **Χ**^2^ tests were applied. Age was considered as continuous variable for correlational analysis and the sample was binned into five age groups (18–30y, 31–40y, 41–50y, 51–60y and 61- years) to perform mean comparisons.

## Additional Information

**How to cite this article**: Beijamini, F. *et al*. Timing and quality of sleep in a rural Brazilian family-based cohort, the Baependi Heart Study. *Sci. Rep.* **6**, 39283; doi: 10.1038/srep39283 (2016).

**Publisher's note:** Springer Nature remains neutral with regard to jurisdictional claims in published maps and institutional affiliations.

## Supplementary Material

Supplementary Information

## Figures and Tables

**Table 1:** Summary of demographic data and from the entire population and sex comparisons.

	Full Sample (n = 1,334)	Female (n = 780)	Male (n = 554)
Age	46.5 ± 16.2	46.8 ± 15.3	47.1 ± 16.7
ESS score	7.4 ± 4.8	7.4 ± 4.8	7.5 ± 4.7
PSQI score***	**4.9 ± 3.2**	**5.4 ± 3.3**	**4.5 ± 2.9**
MEQ score***	**63.6 ± 10.8**	**62.8 ± 10.7**	**64.8 ± 10.9**
Excessive Daytime Sleepiness (ESS > 10)	31.7%	31.9%	31.4%
Poor Sleep Quality (PSQI > 5)***	**34.9%**	**39.9%**	**27.8%**
Bed time (hh:min)	22:32 ± 01:27	22:31 ± 01:26	22:26 ± 01:40
Wake up time*** (hh:min)	**06:17 ± 01:25**	**06:26 ± 01:17**	**06:04 ± 01:34**
Time in bed*** (hh:min)	**07:44 ± 1:29**	**07:52 ± 1:26**	**07:32 ± 1:31**
Self-reported Sleep duration (hh:min ± hh:min)***	**7:06 ± 1:31**	**7:13 ± 1:33**	**6:57 ± 1:27**

The table shows Epworth Sleepiness Scale score (ESS score); Pittsburgh Sleep Quality Index score (PSQI); Morningness-Eveningness Questionnaire score (MEQ); Values presented in means ± standard deviation. Bedtime and wake up time presented in hour ± minutes; Time in bed and Sleep duration presented in hours ± minutes. *** and bold indicate sex difference (p < 0.05) according to Student’s t test or Chi Square comparison. ^a^Epworth questionnaire data from one subject were missing.

**Table 2:** Characteristics of poor and good sleepers.

	Poor sleep quality (N = 465)	Good sleep quality (N = 869)
Chronotype	62.97 (11.16)	63.97 (10.65)
**M-type (N)**	**18**	**79**
**N-type (N)**	**269**	**561**
E-type (N)***	**52**	**73**
Age***	**50.36 (15.74)**	**44.50 (16.74)**
Bedtime***	**22:43 (1:38)**	**22:26 (01:19)**
Wake up time	6:15 (1:38)	6:19 (01:17)
Sleep latency***	**38.9 (39.81)**	**14.24 (10.70)**
Time in bed***	**7:38 (2:19)**	**7:52 (1:13)**
Sleep duration***	**6:11 (1:42)**	**7:36 (01:09)**

Frequency distribution of chronotype were evaluated by Chi-square test. Means ± standard deviations for age, chronotype, bedtime, wake up time, sleep latency, time in bed and sleep duration are reported, Student’s t test was performed to compare poor and good sleepers. Bold and ***indicated statistical significance (p < 0.05).
