# Supplementary material for: Timing and quality of sleep in a rural Brazilian family-based cohort, the Baependi Heart Study
Source: Sci Rep. 2016 Dec 23;6:39283. doi: 10.1038/srep39283 (PMC5180217; doi:10.1038/srep39283)
Supplement: Supplementary Information [file srep39283-s1.doc]

**Timing and quality of sleep in a rural Brazilian family-based cohort, the**

**Baependi Heart Study**

**SUPPLEMENTAL MATERIAL**

Beijamini F.^1,2^, Knutson, K. L^3^., Lorenzi-Filho, G.^4^, Egan, K. J.^5^, Taporoski, T.P.^1^, De Paula L.K.G.^5^, Negrão, A.B.^6^, Horimoto, A.R.V.R.^6^, Duarte N.E.^6^, Vallada, H.^1^, Krieger, J. E.^6^, Pedrazzoli, M.^7^, Pereira, A. C.^5^, von Schantz, M.^1,5,6*^.

^1^ Institute of Psychiatry, University of São Paulo Medical School, São Paulo, SP, Brazil.

^2^ Centre for Health Sciences, State University of Western Paraná, UNIOESTE, Francisco Beltrão, PR, Brazil.

^3^ Department of Medicine, University of Chicago, Chicago IL, USA.

^4^ Sleep Laboratory, Pulmonary Division, Heart Institute (InCor) do Hospital das Clínicas da Faculdade de Medicina da Universidade de São Paulo, SP, Brazil.

^5^ Faculty of Health and Medical Sciences, University of Surrey, Guildford, Surrey, UK

^6^ Laboratory of Genetics and Molecular Cardiology, Heart Institute (InCor), University of São Paulo Medical School, São Paulo, SP, Brazil

^7^ School of Arts, Sciences and Humanities, University of São Paulo, SP, Brazil.

* Corresponding author. Address for correspondence:
Faculty of Health and Medical Sciences, University of Surrey, Guildford, Surrey GU2 7XH, UK, m.von.schantz@surrey.ac.uk

Supplementary data from: **Timing and qualify of sleep in a rural Brazilian family-based cohort, the Baependi Heart Study**.

**Supplemental Figure 1 –Frequency distribution.** Frequency histogram plot for Pittsburgh Sleep Quality Index scores split according to sex.

**
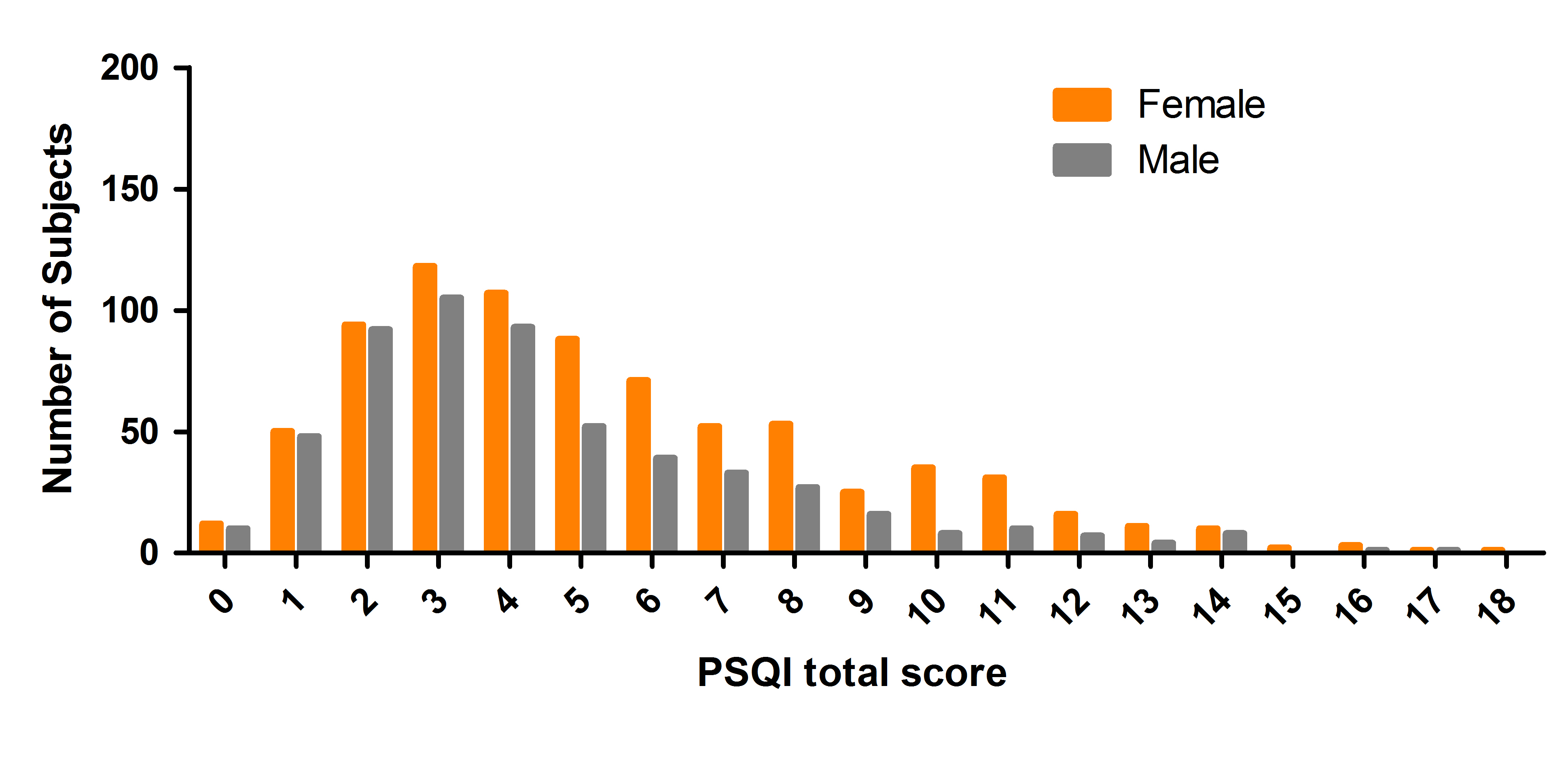
**

**Supplemental Figure 2 – Sleep disturbances categorisation.** Frequency histograms for the 10 categories of sleep disturbances presented of the PSQI questionnaire.

**
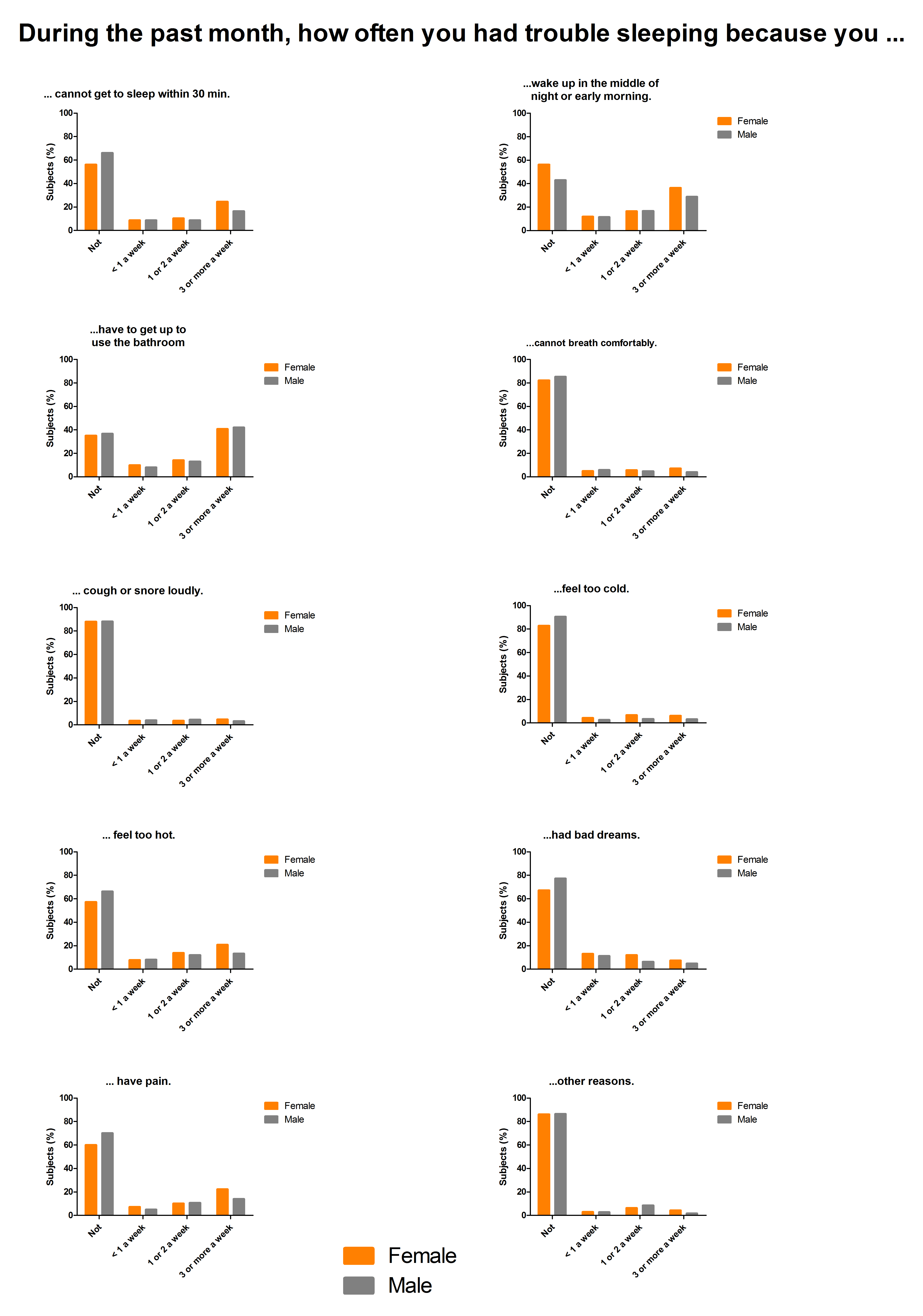
**

**Supplemental Figure 3 – Chronotype categorisation.**

Red line in the middle represents the linear regression. Dotted lines above and below represent the cutt-offs for categorizations of chronotypes as Morning types (M-types), Neither type (N-type) and Evening type (E-type).


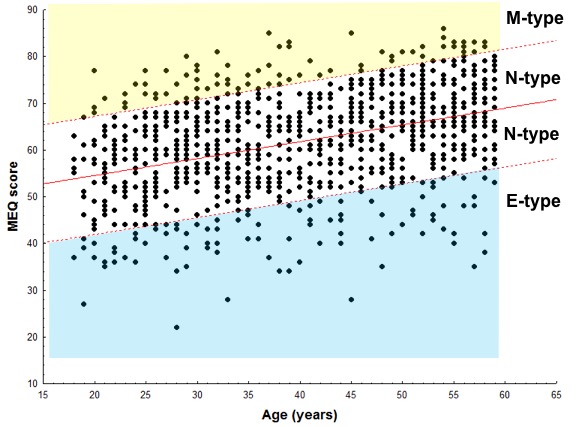


**Supplemental Table 1. Sleep Patterns, sleep quality, sleepiness and chronotype by age groups.**

| **Age bin** | **Sex** | **Bedtime** | **Wake up time** | **Sleep duration** | **MEQ score** | **PSQI score** | **ESS score** | **N** |
| --- | --- | --- | --- | --- | --- | --- | --- | --- |
| 18 – 30y | F | 22:51 ± 07 | 07:20 ± 06 | 480.9 ±7.1 | 56.98 ± 0.77 | 4.25 ± 0.25 | 7.83 ± 0.38 | 156 |
|  | M | 23:23 ± 08 | 06:58 ± 07 | 436.7 ± 8.1 | 56.00 ± 0.87 | 3.89 ± 0.28 | 7.43 ± 0.43 | 120 |
| 31 – 40y | F | 22:39 ± 07 | 06:33 ± 06 | 434.8 ± 7.3 | 59.22 ± 0.78 | 4.9 ± 0.25 | 7.3 ± 0.39 | 150 |
|  | M | 22:35 ± 09 | 06:10 ± 08 | 415.7 ± 9.2 | 61.19 ± 1.00 | 3.93 ± 0.32 | 6.26 ± 0.49 | 92 |
| 41 – 50y | F | 22:29 ± 07 | 06:12 ± 06 | 422.9 ± 7.0 | 63.21 ± 0.75 | 5.45 ± 0.24 | 6.89 ± 0.37 | 162 |
|  | M | 22:07 ± 08 | 06:04 ± 07 | 417.3 ± 8.8 | 65.76 ± 0.94 | 4.27 ± 0.31 | 7.15 ± 0.47 | 103 |
| 51 – 60y | F | 22:29 ± 07 | 06:03 ± 06 | 414.0 ± 7.1 | 64.77 ± 0.77 | 5.98 ± 0.25 | 7.66 ± 0.38 | 155 |
|  | M | 22:07 ± 08 | 05:27 ± 07 | 405.8 ± 8.3 | 69.36 ± 0.90 | 4.97 ± 0.29 | 7.27 ± 0.44 | 114 |
| 61 - up | F | 22:08 ± 07 | 06:01 ± 07 | 411.6 ± 7.1 | 69.69 ± 0.76 | 6.16 ± 0.25 | 7.11 ± 0.38 | 157 |
|  | M | 21:54 ± 08 | 05:40 ± 07 | 413.3 ± 7.8 | 70.76 ± 0.86 | 5.11 ± 0.28 | 8.34 ± 0.42 | 125 |

Table 1. Values presented as means ± standard error. Bedtime and wake up time presented in hour ± minutes; Sleep duration presented in minutes ± minutes.
